# Differentiated nomological networks of internalizing, externalizing, and the general factor of psychopathology (‘*p* factor’) in emerging adolescence in the ABCD study

**DOI:** 10.1017/S0033291720005103

**Published:** 2022-10

**Authors:** Sarah J. Brislin, Meghan E. Martz, Sonalee Joshi, Elizabeth R. Duval, Arianna Gard, D. Angus Clark, Luke W. Hyde, Brian M. Hicks, Aman Taxali, Mike Angstadt, Saige Rutherford, Mary M. Heitzeg, Chandra Sripada

**Affiliations:** Department of Psychiatry, University of Michigan, 4250 Plymouth Rd, Ann Arbor, MI 48109, USA

**Keywords:** ABCD study, emerging adolescence, externalizing, general factor of psychopathology, internalizing, *p* factor

## Abstract

**Background:**

Structural models of psychopathology consistently identify internalizing (INT) and externalizing (EXT) specific factors as well as a superordinate factor that captures their shared variance, the *p* factor. Questions remain, however, about the meaning of these data-driven dimensions and the interpretability and distinguishability of the larger nomological networks in which they are embedded.

**Methods:**

The sample consisted of 10 645 youth aged 9–10 years participating in the multisite Adolescent Brain and Cognitive Development (ABCD) Study. *p*, INT, and EXT were modeled using the parent-rated Child Behavior Checklist (CBCL). Patterns of associations were examined with variables drawn from diverse domains including demographics, psychopathology, temperament, family history of substance use and psychopathology, school and family environment, and cognitive ability, using instruments based on youth-, parent-, and teacher-report, and behavioral task performance.

**Results:**

*p* exhibited a broad pattern of statistically significant associations with risk variables across all domains assessed, including temperament, neurocognition, and social adversity. The specific factors exhibited more domain-specific patterns of associations, with INT exhibiting greater fear/distress and EXT exhibiting greater impulsivity.

**Conclusions:**

In this largest study of hierarchical models of psychopathology to date, we found that *p*, INT, and EXT exhibit well-differentiated nomological networks that are interpretable in terms of neurocognition, impulsivity, fear/distress, and social adversity. These networks were, in contrast, obscured when relying on the a priori Internalizing and Externalizing dimensions of the CBCL scales. Our findings add to the evidence for the validity of *p*, INT, and EXT as theoretically and empirically meaningful broad psychopathology liabilities.

## Introduction

Early adolescence is an important developmental period when biopsychosocial transitions (e.g. school entry, puberty) increase risk for youth psychopathology (Crone & Dahl, 2012; Smetana, Campione-Barr, & Metzger, 2006). Symptoms that emerge during this time are predictive of severe forms of adult psychopathology (Copeland, Shanahan, Costello, & Angold, 2009) and perniciously impact health, wealth, and wellbeing across the life course (Copeland, Wolke, Shanahan, & Costello, 2015; Erskine et al., 2014). However, efforts to identify at-risk youth and develop effective treatments may be hampered by the current categorical schemes of psychiatric diagnosis, which yield troublingly high levels of comorbidity suggesting disorder boundaries drawn incorrectly (Angold, Costello, & Erkanli, 1999; Kessler et al., 2005; Krueger & Markon, 2006), rely on arbitrary thresholds for meeting disorder criteria (Lewinsohn, Shankman, Gau, & Klein, 2004; Rucci et al., 2003), and which may not line up well with neural mechanisms and neurodevelopmental processes (Casey, Oliveri, & Insel, 2014; Cuthbert, 2014).

Alternative, dimensional models of psychopathology have emerged primarily by using factor analytic techniques to identify patterns of covariance across psychiatric symptoms. Initial work defined two correlated dimensions: internalizing (INT), reflecting covariance among depression and anxiety diagnoses, and externalizing (EXT), capturing comorbidity among substance use and delinquent behavior (Achenbach, 1966; Krueger, 1999; Krueger & Markon, 2006). Further work, however, also identified a superordinate dimension, the ‘*p* factor’ (Caspi et al., 2014; Caspi & Moffitt, 2018; Lahey et al., 2012, 2015; Patalay et al., 2015; Smith, Atkinson, Davis, Riley, & Oltmanns, 2020; Tackett et al., 2013), that captures their shared variance. This hierarchical structure reliably emerges across different study designs (Caspi et al., 2014, 2017; Castellanos-Ryan et al., 2016; Lahey et al., 2015; Murray, Eisner, & Ribeaud, 2016; Noordhof, Krueger, Ormel, Oldehinkel, & Hartman, 2015; Patalay et al., 2015; Pettersson, Lahey, Larsson, Lundstroem, & Lichtenstein, 2015; Stochl et al., 2015), across age groups including children (Lahey et al., 2015; Martel et al., 2017; Murray et al., 2016; Neumann et al., 2016; Waldman, Poore, van Hulle, Rathouz, & Lahey, 2016), adolescents (Blanco et al., 2015; Bloemen et al., 2018; Carragher et al., 2016; Castellanos-Ryan et al., 2016; Laceulle, Vollebergh, & Ormel, 2015; Lahey et al., 2008, 2011; Noordhof et al., 2015; Patalay et al., 2015; Stochl et al., 2015; Tackett et al., 2013; Waldman et al., 2016), and adults (Caspi et al., 2014; Lahey et al., 2012; Stochl et al., 2015; Wright & Simms, 2015), and across assessment methods (Blanco et al., 2015; Kotov et al., 2017; Stochl et al., 2015). Recently work also found that subjects' rank-order on *p*, INT, and EXT dimensions are robust across a wide variety of modeling choices (e.g. bifactor models and higher-order factor models; Clark et al., 2020). Despite these trends, controversy lingers about the meaning of these data-driven dimensions: Do they represent real liabilities for broad psychopathological risk, or do they instead perhaps reflect ‘artifactual’ causes (e.g. rater response style, general distress at the time of assessment)?

The validity of hypothesized constructs ultimately depends upon locating them in nomological networks, that is, theoretically coherent pattern of linkages among the constructs, other constructs, and observable variables that accounts for their interrelationships (Cronbach & Meehl, 1955). Prior studies made progress on this front, reporting that the *p* factor is associated with temperament (high negative emotionality, low constraint, high impulsivity; Brandes, Herzhoff, Smack, and Tackett, 2019; Caspi et al., 2014; Castellanos-Ryan et al., 2016; Hankin et al., 2017; Tackett et al., 2013), poor executive function and lower cognitive ability scores (Bloemen et al., 2018; Caspi et al., 2014; Castellanos-Ryan et al., 2016; Hankin et al., 2017; Lahey et al., 2015; Martel et al., 2017; Michelini et al., 2019; Snyder, Friedman, & Hankin, 2019a), worse mental health outcomes (Michelini et al., 2019; Pettersson, Lahey, Larsson, & Lichtenstein, 2018), common genetic loading across psychiatric disorders (Neumann et al., 2016; Selzam, Coleman, Caspi, Moffitt, & Plomin, 2018), structural and functional neural alterations (e.g. reduced grey matter volume) (Alnæs et al., 2018; Elliott, Romer, Knodt, & Hariri, 2018), and socioenvironmental variables including lower birth weight, unsupportive and hostile parenting, and lower socioeconomic status (Carver, Johnson, & Timpano, 2017; Caspi & Moffitt, 2018). In contrast to *p*, less is known about INT and EXT in the context of these hierarchical models, especially bifactor models. INT has been consistently associated with elevation in fear/distress, while associations with other variables (e.g. positive affect; Hankin et al., 2017), neurocognition) have been inconsistent. This could be due to the failure to account for shared variance with the *p* factor. For example, internalizing disorders are associated with reduced cognitive abilities (Levin, Heller, Mohanty, Herrington, & Miller, 2007; Rapport, Denney, Chung, & Hustace, 2001; Snyder, 2013), while studies that remove the variance associated with *p* in a bifactor model have found the remaining INT factor is associated with elevated cognitive abilities (Lahey et al., 2015; Masten et al., 2005; Patalay et al., 2015; Tackett et al., 2013). Research has also been limited on nomological associations of EXT after accounting for *p*. Previous studies linked the externalizing dimension (Beauchaine & McNulty, 2013; Hinshaw, 2002) to psychological deficits, including reduced neurocognition (Bloemen et al., 2018), effortful control (Deutz et al., 2020; Hankin et al., 2017), and socioemotional functioning, as well as environmental risk factors, including low SES, reduced environmental enrichment, and harsh parenting (Beauchaine, Shader, & Hinshaw, 2016). However, most previous work was conducted without simultaneously modeling the *p* factor, and therefore it is unclear if these associations are unique to EXT or driven by *p*.

In the present study, we build upon and extend previous work by examining the nomological networks of the *p* and INT and EXT specific factors in baseline data from the ABCD multisite study (Volkow et al., 2018) of 118 759- to10-year-old emerging adolescents. While recent work by our group and others also has sought to define the structure of psychopathology in this sample (Clark et al., 2020; Michelini et al., 2019; Moore et al., 2020), these studies have focused primarily comparing alternative structural models of the Child Behavior Checklist (CBCL) (Clark et al., 2020; Michelini et al., 2019; Moore et al., 2020) and validated the derived factors focusing on a small number of criterion variables with the goal of comparing the different factors (Clark et al., 2020; Michelini et al., 2019; Moore et al., 2020). The current study will examine the convergent and divergent associations between the general (*p*) and specific (EXT and INT) factors and a comprehensive, multi-domain pool of criterion measures. We focus on delineating the full unique and overlapping profiles of these latent liabilities, as well as comparing their respective nomological network with those that emerge from the original CBCL Internalizing and Externalizing scales. The ABCD study involves a comprehensive assessment battery (Barch et al., 2018), covering demographics, psychopathology, temperament, family history of substance use and psychopathology, socio-environment (school and family environment), and cognitive ability. Multiple assessment modalities are also included, such as youth-, parent-, and teacher-report questionnaires, interviews, and behavioral task performance. Moreover, the ABCD study used a multi-stage probability sampling strategy, and with sample weighting approximates a representative US population sample of 9- and 10-year olds (Garavan et al., 2018), thus improving generalizability (Deutz et al., 2020; Snyder, Young, & Hankin, 2019b). Results from our investigation found that *p*, INT, and EXT exhibit distinct and well-differentiated nomological networks that are readily interpreted in terms of neurocognitive, temperamental, and social factors. These nomological networks were, in contrast, obscured when relying on the a priori internalizing and externalizing dimensions of the CBCL scales.

## Methods

### Participants

The study used data collected from the ABCD Study, a large-scale study of youth aged 9–10 years (*N* = 10 645), recruited from 21 research sites across the USA (Barch et al., 2018; Garavan et al., 2018; Volkow et al., 2018). These data were collected from baseline visits between 1 September 2016 and 15 November 2018. The data used in this report came from ABCD Release 2.01, DOI: 10.15154/1504041. The sample was roughly gender-balanced (47.6% female) with a mean age of 9.93 years (s.d. = 0.62 years). Around half (50.7%) of the sample was White, with the remaining participants identifying themselves as Hispanic (19.7%), African-American (14.5%), Other/Multi-racial (9.8%), or Asian (2.1%). Data on ethnicity was missing for 3.2% of the sample. Approximately two-thirds of youth (65.5%) came from households in which the parents were married. Most parents (83.0%) reported at least some college and most households reported an annual income of at least $50 000 (62.6%).

### Measures

The *Child Behavior Checklist* (Achenbach et al., 2000) was used to compute the standard Internalizing Problems and Externalizing Problems composite scales. The Withdrawn, Somatic Complaints, and Anxious/Depressed scales all contribute to higher order Internalizing Problems composite scale, while the Delinquent Behavior and Aggressive Behavior scales contribute to a higher order Externalizing Problems composite scale. The Internalizing and Externalizing Problems scales were *t*-scored by gender.

### *p*, INT, and EXT

A general *p* factor and orthogonal EXT and INT factors were modeled using the parent-rated CBCL (age 6–18; Achenbach et al., 2000). The *p*, EXT, and INT factor scores used in subsequent analyses were derived by fitting a bifactor model to the 8 CBCL scales (Withdrawn, Somatic Complaints, Anxious/Depressed, Social Problems, Thought Problems, Attention Problems, Delinquent Behavior, and Aggressive Behavior). In this model, there was a general *p* factor that all scales loaded onto (average scale loading on *p* = 0.69), and two specific factors: EXT and INT (average scale loading on sub-factors = 0.43). The EXT specific factor included the Delinquent and Aggressive Behaviors scales, while the INT-specific factor included the Withdrawn, Somatic Complaints, and Anxious/Depressed scales. This model fit well was based on conventional fit thresholds (χ^2^ = 747.73, df = 16, *p* < 0.001; RMSEA = 0.062; CFI = 0.985; TLI = 0.974; SRMR = 0.015) and was chosen for its good model fit and theoretical interpretability. Across a variety of alternative specifications of hierarchical models of psychopathology in ABCD, the resulting *p*, INT, and EXT factors that emerge are broadly similar (Clark et al., 2020), rendering decisions about which specific modeling strategy to adopt less consequential.

### Family history variables

*Family History of Substance Problems* was computed from the ABCD's Family History Assessment (Barch et al., 2018). A threshold is established for a family member counting as an affected case based on the number of serious problems that person has had due to alcohol use and substance use. The following coding was used: 0 = neither parent met the threshold; 1 = one or more parents met the threshold.

*Family History of Psychopathology* Based on previously published protocols (Milne et al., 2008, 2009), a family history composite score was constructed from responses for ABCD's Family History Assessment (Barch et al., 2018; see Supplement for further description).

### Area deprivation index

Area deprivation index (ADI) scores were available for each participant for up to three residences. A weighted average of ADI scores was computed based on months lived at each residence.

### The Diagnostic and Statistical Manual of Mental Disorders, Fifth Edition (DSM-5) psychopathology

The *Kiddie-Structured Assessment for Affective Disorders and Schizophrenia for DSM-5* (KSADS-5) is a structured, diagnostic interview that was administered to parents via computer in reference to their child (Barch et al., 2018; Kobak, Kratochvil, Stanger, & Kaufman, 2013, see online Supplementary Methods for detailed description of coding).

The *Prodromal Questionnaire- Brief Version* (PQ-B; Loewy, Bearden, Johnson, Raine, and Cannon, 2005) is a youth-report measure designed to index subclinical prodromal psychosis risk phenotypes.

The *Brief Problem Monitoring Form* (BPM; Piper, Gray, Raber, and Birkett, 2014) is an abbreviated, 18-item version of the CBCL that was sent to teachers for completion. Completion rate was 35% (*N* = 4495). This measure produces three *t*-scores regarding youth psychopathology: Internalizing Problems, Externalizing Problems, and Attention Problems.

### Trait measures

The behavioral inhibition system/behavioral activation system (*BIS/BAS*) measure (Pagliaccio et al., 2016) is a 24-item scale designed to assess three facets of behavioral activation, reflecting positive affect: Drive, Fun Seeking, and Reward Responsiveness, and a Behavioral Inhibition scale, indexing sensitivity to punishment.

A 20-item youth short version of the Urgency, Premeditation (lack of), Perseverance (lack of), Sensation Seeking, Positive Urgency, Impulsive Behavior Scale (*UPPS-P)*, developed for the use in the ABCD study (Barch et al., 2018) was administered via self-report at baseline to index trait impulsivity yielding five subscales: Negative Urgency, Positive Urgency, Lack of Perseverance, Lack of Planning, and Sensation Seeking.

### School environment

Youth reported on *School Risk and Protective Factors* (*SRPF*) to assess their connection to the school environment (Zucker et al., 2018). This measure was taken from the PhenX Toolkit, yielding three subscales: School Environment, School Involvement, and School Disengagement scale.

### Social functioning

Parents and youth reported on the prosocial behavior of the youth using the *Prosocial Behavior Scale*, which is a 3-item scale formed from the ‘Strengths and Difficulties Questionnaire’ (SDQ; Goodman, 2001).

### Family environment

Parent and child both rated the quality of the family environment with the Family Conflict subscale from the PhenX Toolkit *Family Environment Scale* (Zucker et al., 2018).

Youth reported on their perceived level of parental monitoring using the *Parental Monitoring Survey*, a scale developed to assess parents' efforts to keep track of their child's whereabouts (Zucker et al., 2018).

### Neurocognition

*General Neurocognition* (GN) scores were computed by fitting a bifactor model to behavioral tasks from the NIH toolbox, the Rey Auditory Verbal Learning Task, the WISC-V, and the ‘Little Man’ task (Sripada, Taxali, Angstadt, & Rutherford, 2020). Exploratory factor analyses suggested that three broad factors characterized these tasks, corresponding to memory, speed/flexibility, and reasoning; in the bifactor model, these three factors served as the specific factors. The bifactor model fit well by conventional standards: χ^2^ = 443.16, df = 34, *p* < 0.001; RMSEA = 0.032; CFI = 0.990; TLI = 0.983; SRMR = 0.017 (West, Taylor, & Wu, 2012). GN factor scores (i.e. scores for the general factor in the bifactor model) were generated using maximum a posteriori scoring (MacCallum, 2009).

### Data analytic strategy

All analyses were performed using R. Data were weighted to correspond to the American Community Survey proportions and analyses accounted for clustering within the collection site and family (Heeringa & Berglund, 2020)^†^^1^. All SEM analyses were performed with *laavan* (open-source code at https://github.com/SripadaLab/ABCD_nomological_networks). Factor scores were estimated in factor models as described above, and regressions involving these factors were concurrently performed (in the latent space), controlling for the following covariates: participant sex, race, parent education, parent marital status, household income. Associations between CBCL Internalizing Problems and Externalizing Problems *t*-scores (gender normed) with the same set of independent variables were examined using multilevel models, with the same covariates excluding sex. When examining associations with demographic variables, all demographic variables were entered into one model. Given the large sample size, we chose to use the conservative alpha level a False Discovery Rate Benjamini-Hochburg corrected *p* < 0.001 to determine significance.

## Results

**Fig. 1.**
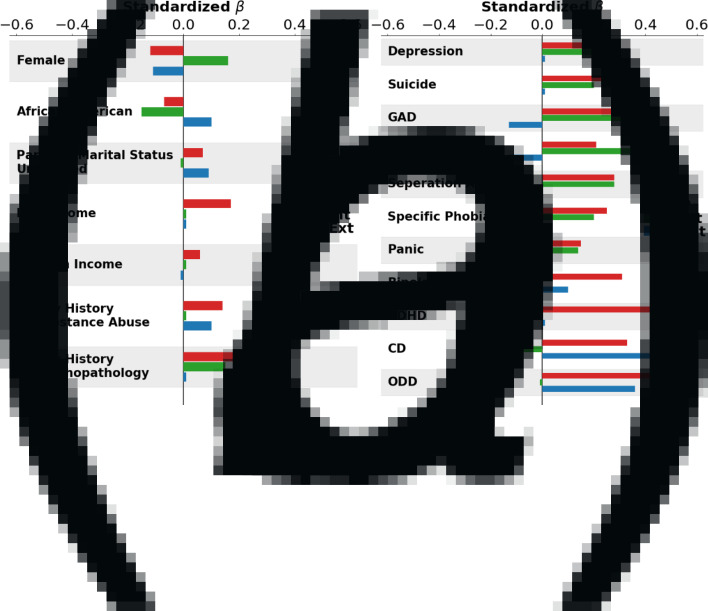
Visualization of associations between *p*, INT, and EXT and independent variables. (a) Standardized beta weights with demographic variables; (b) Standardized beta weights with KSAD Diagnoses.

**Fig. 2.**
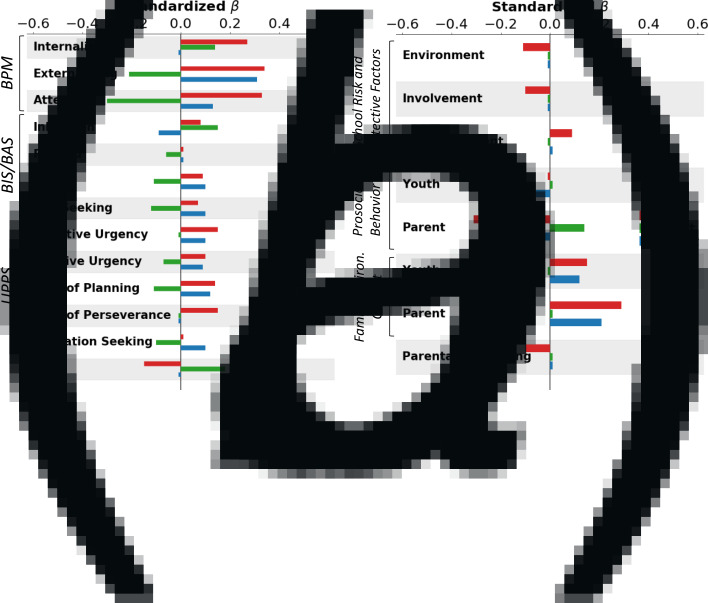
Visualization of associations between *p*, INT, and EXT and independent variables. (a) Standardized beta weights with teacher-rated psychopathology (BPM), trait measures (BIS/BAS and UPPS), and general neurocognition (GN); (b) Significant associations with school environment (School Risk and Protective Factors), Prosocial Behavior (youth and parent report), Family Conflict (youth and parent report), and Parental Monitoring (youth report).

*p*. Regarding demographics and family history (Table 1, Fig. 1*a*) *p* scores were higher for males relative to females, white relative to African-American, Hispanic, and Asian youth, children in lower income households, children with unmarried parents, and children with a higher family loading of mental health and substance use problems. *p* factor scores were significantly associated with all KSADS diagnoses, prodromal psychotic symptoms, and teacher ratings of Internalizing, Externalizing, and Attention problems (Table 2, Fig. 1*b*). Regarding associations with personality traits (Table 2, Fig. 2*a*) *p* scores were associated with higher BIS and BAS Drive and Fun Seeking scale scores as well as higher Negative Urgency, Positive Urgency, Lack of Planning and Lack of Perseverance scores. In addition, *p* was associated with lower scores on GN (Table 2, Fig. 2*a*). Lastly, *p* factor scores were associated with the worse school environment, lower school involvement, greater school disengagement, less prosocial behavior, more family conflict, and less parental monitoring (Table 3, Fig. 2*b*).

**Table 1.** Associations between demographic variables and *p*, Internalizing (INT), and Externalizing (EXT) factor scores

	*p*	INT	EXT
	*β* (95% CI)	*β* (95% CI)	*β* (95% CI)
Sex			
Female	−0.12* (−0.14–−0.09)	0.16* (0.13–0.19)	−0.11* (−0.14–−0.08)
Race/Ethnicity			
African-American	−0.07* (−0.10–−0.04)	−0.15* (−0.19–10.12)	0.10* (0.05–0.14)
Hispanic	−0.06* (−0.09–−0.03)	0.05 (0.01–0.09)	−0.06 (−0.10–−0.01)
Asian	−0.04* (−0.07–−0.02)	−0.04 (−0.07–−0.02)	−0.01 (−0.03–0.02)
Other	0.02 (−0.01–0.05)	−0.05 (−0.08–−0.02)	0.05 (0.01–.10)
Parental marital status			
Unmarried	0.07* (0.03–0.10)	−0.01 (−0.05–0.03)	0.09* (0.05–0.13)
Highest parent education			
HS or equivalent degree	0.01 (−0.04–0.06)	0.02 (−0.04–0.07)	−0.03 (−0.10–0.05)
Associates or occupational	0.03 (−0.02–0.08)	0.12* (0.06–0.18)	−0.07 (−0.14–0.02)
Some college	0.04 (−0.02–0.09)	0.10 (0.03–0.16)	−0.06 (−0.14–0.02)
College degree	0.00 (−0.07–0.06)	0.16* (0.08–0.23)	−0.13 (−0.22–−0.04)
Masters or professional degree	−0.04 (−0.10–0.02)	0.20* (0.12–0.27)	−0.15 (−0.23–−0.06)
Household income			
<$50 000	0.17* (0.14–0.21)	0.05 (−0.01–0.10)	0.07 (0.01–0.12)
$50 000–$100 000	0.06* (0.03–0.08)	0.04 (0.00–0.07)	−0.01 (−0.04–0.02)
Age	−0.02 (−0.04–0.00)	0.05 (0.02–0.08)	0.02 (−0.02–0.05)
Family history of substance use problems	0.14* (0.10–0.17)	0.02 (−0.02–0.06)	0.10* (0.06–0.15)
Family history of psychopathology	0.28* (0.25–0.31)	0.15* (0.11–0.19)	0.01 (−0.03–0.05)
Area deprivation index	−0.05 (−0.43–0.33)	0.00 (−0.22–0.21)	−0.04 (−0.28–0.19)

*Note*. Significance (*p < 0.001) determined from latent variable model where associations between *p*, INT, and EXT and all demographic variables were weighted to correspond to American Community Survey proportions and accounted for in the same model. Separate models were used to examine associations between *p*, INT, and EXT and Family History variables and Area Deprivation Index. Comparison groups for *β*'s are Male (Sex); White (Race/Ethnicity); Married (Parental Marital Status); Less than High School Education (Highest Parental Education); <$100 000 (Household Income); No Parents with Substance Use Problems (Family History of Substance Use Problems); all other variables are continuous. Bolded values |*β*| ⩾ 0.20, denote at least a medium effect size.

**Table 2.** Associations with psychopathology, trait measures, and neurocognition

	*p*	INT	EXT	Internalizing problems	Externalizing problems
	*β* (95% CI)	*β* (95% CI)	*β* (95% CI)	*β* (95% CI)	*β* (95% CI)
KSAD Diagnoses					
Depression	0.29* (0.25–0.33)	0.21* (0.16–0.26)	0.00 (−0.05–0.05)	0.23* (0.21–0.25)	0.20* (0.18–0.21)
Suicide/Self-harm behaviors	0.36* (0.32–0.40)	0.20* (0.16–0.25)	0.07 (0.02–0.12)	0.30* (0.27–0.33)	0.29* (0.26–0.32)
Generalized anxiety	0.35* (0.31–0.39)	0.39* (0.34–0.45)	−0.13* (−0.18–−0.09)	0.34* (0.31–0.37)	0.22* (0.19–0.25)
Social anxiety	0.21* (0.17–0.25)	0.35* (0.30–0.41)	−0.13* (−0.17–−0.09)	0.25* (0.21–0.28)	0.12* (0.10–0.15)
Separation anxiety	0.28* (0.25–0.32)	0.28* (0.23–0.32)	−0.07 (−0.11–−0.03)	0.31* (0.28–0.33)	0.22* (0.19–0.24)
Specific phobia	0.25* (0.23–0.28)	0.20* (0.16–0.23)	−0.05 (−0.09–−0.02)	0.26* (0.24–0.28)	0.19* (0.17–0.21)
Panic/Agoraphobia	0.15* (0.11–0.20)	0.14* (0.09–0.19)	−0.02 (−0.07–0.03)	0.13* (0.09–0.16)	0.10* (0.07–0.13)
Bipolar	0.31* (0.27–0.35)	−0.02 (−0.06–0.03)	0.10* (0.05–0.15)	0.20* (0.17–0.22)	0.25* (0.22–0.28)
ADHD	0.52* (0.50–0.55)	−0.21* (−0.25–−0.17)	0.08 (0.04–0.12)	0.29* (0.27–0.31)	0.40* (0.37–0.42)
Conduct disorder	0.33* (0.28–0.38)	−0.15* (−0.20–−0.10)	0.43* (0.36–0.49)	0.16* (0.13–0.20)	0.31* (0.27–0.36)
ODD	0.47* (0.44–0.50)	−0.05 (−0.09–−0.01)	0.36* (0.32–0.40)	0.29* (0.26–0.31)	0.49* (0.46–0.52)
Prodromal questionnaire	0.14* (0.01–0.18)	−0.03 (−0.06–0.00)	0.04 (0.00–0.08)	0.09* (0.07–0.11)	0.12* (0.10–0.14)
Brief problems monitor					
Internalizing	0.27* (0.23–0.32)	0.14* (0.08–0.20)	−0.05 (−0.10–0.01)	0.20* (0.18–0.23)	0.17* (0.15–0.20)
Externalizing	0.34* (0.29–0.39)	−0.21* (−0.27–−0.14)	0.31* (0.25–0.37)	0.13* (0.10–0.16)	0.35* (0.32–0.38)
Attention	0.33* (0.28–0.38)	−0.30* (−0.37–−0.23)	0.13* (0.06–0.20)	0.09* (0.06–0.13)	0.28* (0.24–0.31)
BIS/BAS					
BIS	0.08* (0.05–0.10)	0.15* (0.12–0.18)	−0.09* (−0.12–−0.05)	0.10* (0.08–0.11)	0.02 (0.00–0.04)
BAS- Reward response	0.03 (0.00–0.05)	−0.06* (−0.10–−0.03)	0.04 (0.00–0.07)	−0.02 (−0.04–−0.01)	0.02 (0.00–0.04)
BAS- Drive	0.09* (0.06–0.11)	−0.11* (−0.15–−0.08)	0.10* (0.06–0.13)	0.00 (−0.02–0.02)	0.09* (0.07–0.11)
BAS- Fun seeking	0.07* (0.05–0.10)	−0.12* (−0.15–−0.08)	0.10* (0.06–0.13)	−0.02 (−0.04–0.00)	0.06* (0.05–0.08)
UPPS					
Negative urgency	0.15* (0.12–0.17)	−0.02 (−0.06–0.01)	0.10* (0.06–0.14)	0.08* (0.06–0.10)	0.16* (0.14–0.18)
Positive urgency	0.10* (0.07–0.12)	−0.07* (−0.10–−0.04)	0.09* (0.06–0.13)	0.03* (0.02–0.05)	0.11* (0.10–0.13)
Lack of planning	0.14* (0.12–0.17)	−0.11* (−0.14–−0.08)	0.12* (0.08–0.16)	0.05* (0.03–0.07)	0.16* (0.14–0.18)
Lack of perseverance	0.15* (0.12–0.18)	−0.03 (−0.06–0.00)	−0.02 (−0.05–0.02)	0.08* (0.07–0.10)	0.11* (0.09–0.13)
Sensation seeking	0.01 (−0.01–0.04)	−0.10* (−0.14–−0.07)	0.10* (0.07–0.14]	−0.04* (−0.06–−0.02)	0.04* (0.03–0.06)
General neurocognition	−0.15* (−0.18–−0.12)	0.18* (0.14–0.22)	−0.05 (−0.10–0.00)	−0.03 (−0.05–−0.01)	−0.13* (−0.15–−0.11)

*Note*. Standardized betas from regression analyses with *p*, INT, and EXT and include the following covariates: participant sex, race, parent education level, marital status, and household income. For CBCL Scale analyses standardized betas are from separate multilevel models (MLMs) and sex was excluded as a covariate as scales were t-scored by sex. Analyses were weighted to correspond to American Community Survey proportions and clustered by testing site and family ID. ADHD = Attention Deficit/Hyperactivity Disorder; ODD = Oppositional Defiant Disorder. * False Discovery Rate corrected *p* < 0.001; bolded values |*β*|⩾ 0.20, denote at least a medium effect size.

**Table 3.** Associations with social environment measures

	*p*	INT	EXT	Internalizing problems	Externalizing problems
	*β* (95% CI)	*β* (95% CI)	*Β* ( 95% CI)	*β* (95% CI)	*β* (95% CI)
School risk and protective factors					
School environment	−0.11* (−0.14–−0.08)	−0.03 (−0.06–0.00)	−0.03 (−0.08–0.01)	−0.10* (−0.11–−0.08)	−0.09* (−0.11–−0.07 )
School involvement	−0.10* (−0.13–−0.07)	−0.01 (−0.05–0.02)	−0.01 (−0.06–0.03)	−0.10* (−0.11–−0.08)	−0.11* (−0.13–−0.10)
School disengagement	0.09* (0.06–0.11)	−0.04 (−0.07–0.00)	0.04 (0.01–0.08)	0.06 (0.05–0.08)	0.11* (0.09–0.13)
Prosocial behavior					
Youth report	−0.04 (−0.07–−0.02)	0.03 (0.00–0.06)	−0.06* (−0.09–−0.02)	−0.05* (−0.07–−0.03)	−0.09* (−0.11–−0.07)
Parent report	−0.31* (−0.34–−0.28)	0.14* (0.10–0.17)	−0.26* (−0.31–0.22)	−0.16* (−0.18–−0.14)	−0.33* (−0.33–−0.31)
Family environment: conflict					
Youth report	0.15* (0.12–0.17)	−0.03 (−0.07–0.00)	0.12* (0.08–0.16)	0.08* (0.06–0.10)	0.14* (0.12–0.16)
Parent report	0.29* (0.27–0.32)	0.03 (0.00–0.07)	0.21* (0.17–0.26)	0.21* (0.19–0.23)	0.31* (0.29–0.33)
Parental monitoring survey	−0.10* (−0.13–−0.07)	0.02 (−0.01–0.06)	0.01 (-0.03–0.05)	−0.08* (−0.09–−0.06)	−0.08* (−0.10–−0.06)

*Note*. Standardized betas from regression analyses with *p*, INT, and EXT and include the following covariates: participant sex, race, parent education level, marital status, and household income. For CBCL Scale analyses standardized betas are from separate multilevel models (MLMs) and sex was excluded as a covariate as scales were *t*-scored by sex. Analyses were weighted to correspond to American Community Survey proportions and clustered by testing site and family ID. * False Discovery Rate corrected *p* < 0.001; bolded values |*β*| ⩾ 0.20, denote at least a medium effect size.

INT. When examining associations between demographic and family variables and INT factor scores (Table 1, Fig. 1*a*), INT scores were higher for females compared to males and lower for African-American youth compared to white youth. INT factor scores were also higher for youth from households with higher levels of parental education and with a higher family loading of mental health problems. Regarding associations with psychopathology, INT factor scores were higher for individuals with depression, suicide/self-harm, anxiety disorders, and teacher ratings of internalizing problems (Table 2, Fig. 1*b*). INT scores were significantly lower for individuals with an attention deficit hyperactivity disorder (ADHD) diagnosis, consistent with a negative association with teacher ratings of EXT and attention problems. INT factor scores were significantly associated with higher BIS and lower scores on all BAS facet scales, as well as lower Positive Urgency, Lack of Planning, and Sensation Seeking scores, reflecting a more emotionally and behaviorally constrained personality (Table 2, Fig. 2*a*). INT scores were also associated with higher scores on GN. Lastly, higher INT factor scores were associated with more family conflict per parent report (Table 3, Fig. 2*b*).

EXT. When examining associations between demographic and family history variables and EXT factor scores (Table 1, Fig. 1*a*), EXT scores were higher in males compared to females, African-American compared to white youth, and youth with unmarried parents. EXT factor scores were also higher for individuals who had at least one parent with a substance use problem. Regarding DSM-5 psychopathology (Table 2, Fig. 1*b*), youth diagnosed with conduct disorder (CD), oppositional defiant disorder, or bipolar disorder scored higher on the EXT factor than those without diagnoses. These findings are consistent with positive associations between EXT factor scores and teacher ratings of externalizing problems. Showing discriminant associations, youth that was diagnosed with generalized or social anxiety scored lower on the EXT factor than those that did not meet criteria for diagnosis. EXT factor scores were associated with lower BIS scores, higher BAS-Drive and Fun Seeking scores, and higher Negative Urgency, Positive Urgency, Lack of Planning, and Sensation Seeking scores (Table 2, Fig. 2*a*). Lastly, EXT factor scores were less prosocial behavior and more family conflict.

### CBCL Internalizing Problems and Externalizing Problems

In contrast to findings for INT (which accounts for the variance due to *p*), the CBCL Internalizing Problems scale showed a relatively non-specific pattern of associations, with positive associations with mood and anxiety disorders as expected, but also with CD, ODD, and ADHD (Table 2). In addition, the Internalizing Problems scores were associated with higher levels of trait impulsivity, as evidenced by positive associations with UPPS subscales, counterintuitive to conceptualizations of internalizing proneness as reflecting high levels of constraint. Furthermore, in contrast to EXT, the CBCL Externalizing Problems scale again showed a non-specific pattern of associations with nearly all DSM-5 psychopathology, including, somewhat counterintuitively, internalizing disorders such as depression and anxiety disorders. Scores on the Externalizing Problems scale were also associated with higher scores on the prodromal psychosis scale, teacher-reported internalizing, externalizing, and attention problems, impulsivity, and lower scores on GN.

## Discussion

We delineated the nomological networks of three major dimensions that emerge in hierarchical models of psychopathology, the general factor of psychopathology (‘*p* factor’) and specific INT and EXT factors, in a large, diverse sample of emerging adolescents. We found *p*, INT, and EXT were each associated with nomological networks readily distinguishable along four axes: neurocognition (*p* negatively associated, INT positively associated, EXT unrelated), fear/distress emotions (*p* and INT positively associated), impulsivity (*p* and EXT positively associated), and social adversity (*p* and EXT positively associated). Notably, these distinct nomological networks were obscured when looking at the CBCL Internalizing and Externalizing Problems scores without accounting for their substantial overlap (i.e. without accounting for a shared variance from a superordinate *p* factor). Overall, our results show that three major dimensions that emerge from hierarchical models of psychopathology form clearly distinguishable and readily interpretable nomological networks, adding to the evidence that they help to ‘carve nature at its joints’.

Our results highlight distinct cognitive and temperamental profiles associated with *p*, INT, and EXT, perhaps offering insights into the psychological and neural mechanisms that drive these broad liabilities. As a summary, Table 4 highlights associations between *p*/INT/EXT and four dimensions of importance: GN, two emotion/impulse dimensions: a fear/distress dimension (indexed by BIS and KSADS symptom profile), an impulsivity dimension (indexed by UPPS and BAS subscales and KSADS symptom profile), and a social adversity dimension (indexed by School Risk and Protective Factors and Family Conflict scales). *p*'s position in this space reflects more pervasive alterations involving low neurocognition as well as elevations in both fear/distress and impulsivity profiles in the context of high social and environmental adversity. This might reflect a state in which there are both globally elevated emotions – negative and positive – and impulses as well as reduced executive control capacities to modulate these impulses (Carver et al., 2017; Caspi & Moffitt, 2018; Deutz et al., 2020). EXT and INT, in contrast, reflect domain-specific alterations: elevated impulsivity profile in EXT and elevated inhibition profile in INT. INT is in addition associated with elevated neurocognition, which in turn might help explain INT's complex pattern of associations. For example, it is possible that higher neurocognition contributes to higher fear/distress symptoms due to greater prospection and associated ruminative worry (Penney, Miedema, & Mazmanian, 2015), but at the same time, it is protective for attention problems (i.e. lower BPM Attention and ADHD) due to enhanced cognitive control. Overall, while our pattern of findings is consistent with previous studies (Bloemen et al., 2018; Brandes et al., 2019; Carver et al., 2017; Castellanos-Ryan et al., 2016; Deutz et al., 2020; Hankin et al., 2017; Martel et al., 2017; Michelini et al., 2019; Moore et al., 2020; Tackett et al., 2013), this study is among the first to position these liabilities for psychopathology along these cognitive, temperament, and environmental continua simultaneously, providing novel insights into their interrelationships.

**Table 4.** Summary of results

	*p*	INT	EXT
Social adversity	Elevated	Unrelated	Elevated
Impulsivity	Elevated	Reduced	Elevated
Fear/Distress	Elevated	Elevated	Reduced
General neurocognition	Reduced	Elevated	Unrelated

The *p* factor is conceptualized as a broad liability to all forms of prevalent psychiatric symptomatology. Interestingly, the *p* factor's nomological network was similarly extensive across domains and included: a higher load of family history of psychopathology (consistent with a high heritability of *p*; Allegrini et al., 2020; Selzam et al., 2018); reduced family income; worse family, school, and neighborhood environment; reduced cognitive ability; and elevated emotional responses (including fear/distress emotions and impulsivity). In addition, *p* was associated with a broad array of KSADS diagnoses. Importantly, *p*'s nomological network was established through instruments anchored in youth-, parent-, and teacher-report as well as task-based behavioral performance, suggesting rater-style alone cannot explain the breadth of *p*-associated risk variables. In contrast to *p*, nomological associations of the specific INT and EXT were generally more domain-specific. INT and EXT diverged from each other in demographic associations, with males and African-American youth having higher EXT, and females having higher INT. These distinct patterns of nomological associations for *p*, INT, and EXT raise additional questions about their ultimate drivers (e.g. environment, genes, environment × gene interactions) and perhaps identify targets for prevention efforts that can impact the emergence of broad psychopathology.

It is notable that the nomological networks of *p*, INT, and EXT were obscured when examining a priori Internalizing and Externalizing Problems scales, which demonstrated non-specific associations when not controlling for common variance due to *p*. That is, the dimensions derived from the bifactor structural model of psychopathology yielded improved convergent and divergent validity compared to the standard CBCL-based measures of Internalizing and Externalizing. For example, internalizing is broadly conceptualized in terms of elevated inhibition and threat sensitivity, while externalizing is conceptualized in terms of disinhibition and reduced sensitivity to threats (Krueger, McGue, & Iacono, 2001; Nigg, 2017). Yet, CBCL Internalizing scores are positively associated with CD, while CBCL Externalizing scores were counterintuitively positively associated with multiple anxiety disorders. In contrast, the INT and EXT factors that control for the common variance of *p* exhibit the relationships predicted by prior theory (INT inversely associated with CD; EXT inversely associated anxiety disorders). More broadly, INT and EXT exhibit sharply differentiated, interpretable relationships with a host of variables (teacher-rated externalizing and attention problems, BAS-Drive, GN) that are, in contrast, not visible with the CBCL Internalizing and Externalizing Problems scales. Previous research has raised a concern about the ‘perils of partialing’ (Lynam, Hoyle, & Newman, 2006), wherein well-defined and interpretable scales can sometimes lose their original interpretation when portions of their variance are partialed away. Results from this study suggest that partialing via a bifactor model is not a ‘peril’ in this case. Instead, here partialing yields more interpretable INT, EXT, and *p* constructs, which exhibit more differentiated nomological networks that are better aligned with prior theory.

A striking finding in our results is that nomological associations of INT often diverged from those of the *p* factor. For example, *p* was related to higher attention problems, higher behavioral activation and impulsivity, and lower GN, while INT was associated with fewer attention problems, lower behavioral activation, and higher GN. This observation is noteworthy because the *p* factor represents a liability to all prevalent psychiatric symptoms, which includes internalizing spectrum symptoms. Put another way, our results show that two underlying factors contribute to observed fear and distress symptoms measured by the CBCL: a broad liability *p* factor and a narrow liability INT factor. But importantly, these two liabilities are embedded in highly differentiated, and in several cases, highly divergent, nomological networks. It is notable that the literature on anxiety and depression are similarly mixed, with some studies linking these internalizing disorders to variables associated with *p* (e.g. lower cognitive abilities; Levin *et al*., 2007; Rapport *et al*., 2001; Snyder, 2013), while other studies link them to variables associated with INT (e.g. higher cognitive abilities; Karpinski, Kolb, Tetreault, and Borowski, 2018; Penney et al., 2015). Results of the current study serve to resolve some of these tensions by showing that observed internalizing symptoms likely reflect equifinality wherein distinct underlying liabilities (i.e. *p*-based liability *v.* INT-based liability) lead to similar clinical presentations (i.e. internalizing symptoms). However, future work replicating these findings to confirm their robustness and using person-centered analyses are needed to fully resolve these findings.

Some limitations of the current work warrant mention. First, our hierarchical model of psychopathology was derived from the parent-report CBCL. While studies show parent-report on youth psychopathology is valid at this age; obtaining reports from multiple informants on psychopathology has been found to improve associations with relevant outcomes (Clark, Durbin, Hicks, Iacono, & McGue, 2017). In future waves of ABCD data collection, the youth themselves will complete the CBCL which will strengthen the model estimates of *p*, INT, and EXT. Second, our study captures nomological networks among 9- and 10-year-olds. There is some evidence for the rank-order stability of the *p* factor and specific factors across adolescence (Castellanos-Ryan et al., 2016; McElroy, Belsky, Carragher, Fearon, & Patalay, 2018; Snyder, Young, & Hankin, 2017), but follow-up investigations in future waves of ABCD data are needed to confirm the stability of the observed networks across time.

In sum, the current study demonstrates distinct, divergent, and interpretable patterns of nomological associations for *p*, INT, and EXT in pre-adolescents in the racially and economically diverse ABCD sample. These findings set the stage for future studies in the ABCD sample, leveraging longitudinal waves of data to trace the progression of psychopathology through adolescence, and leveraging multi-modal data to extend the nomological networks described here to encompass biological variables including genes and neurocircuitry.

## Footnotes

† The notes appear after the main text.

## Notes

1 Parallel analyses were run on unweighted data and results were not appreciably different.

## Supplementary material

For supplementary material accompanying this paper visit https://doi.org/10.1017/S0033291720005103. click here to view supplementary material
