# Supplementary material for: Differentiated nomological networks of internalizing, externalizing, and the general factor of psychopathology (‘p factor’) in emerging adolescence in the ABCD study
Source: Psychol Med. 2021 Jan 14;52(14):3051–61. doi: 10.1017/S0033291720005103 (PMC9693677; doi:10.1017/S0033291720005103)
Supplement: Supplementary file 1 [file S0033291720005103sup001.docx]

**Supplemental Methods.**

Family History of Psychopathology Using responses from ABCD’s Family History Assessment [64], responses for the eight categories of psychopathology problems (e.g., depression, mania, ever psychiatrically hospitalized) were tabulated separately for 1st degree relatives (mother, father, full siblings) and second-degree relatives (i.e., everyone else including aunts and uncles, grandparents, etc.). A weighted sum score was computed as follows: first degree cases + 0.5 * second degree cases. This number was then divided by the total score for the given subject, given the relations present in their own family tree. These proportion scores were calculated for each category (e.g., depression) and then summed across categories.

KSAD. Parents completed all modules of the KSADS-5 with the exception of the enuresis, encopresis and selective mutism modules (Barch et al., 2018). KSAD diagnoses were collapsed into the following categories: Depression (coded 1 if met criteria for any past, present Major Depressive Disorder, Persistent Depressive Disorder, Unspecified Depressive Disorder); Suicide/Self-Harm Behaviors (coded 1 if met criteria for any past or present suicide ideation, suicide attempt, or self-injurious behavior); Generalized Anxiety (coded 1 if met criteria for any past, present, or other specified Generalized Anxiety Disorder); Social Anxiety (coded 1 if met criteria for any past, present, or other specified Social Anxiety Disorder); Separation Anxiety (coded 1 if met criteria for any past, present, or other specified Separation Anxiety Disorder); Specific Phobia (coded 1 if met criteria for any past or present Specific Phobia); Panic/Agoraphobia (coded 1 if met criteria for any past, present, or other specified Panic Disorder or Agoraphobia); Bipolar (coded 1 if met criteria for any past, present, or unspecified Bipolar II or Bipolar II Disorder); ADHD (coded 1 if met criteria for past, present, or unspecified ADHD diagnosis); Conduct Disorder (coded 1 if met criteria for past, present Conduct Disorder diagnosis); Oppositional Defiant Disorder (coded 1 if met criteria for past, present Oppositional Defiant Disorder diagnosis).
